# International training programs on eating disorders for professionals, caregivers, and the general public: A scoping review

**DOI:** 10.1186/s40337-015-0066-y

**Published:** 2015-08-14

**Authors:** Myra Piat, Alexis Pearson, Judith Sabetti, Howard Steiger, Mimi Israel, Shalini Lal

**Affiliations:** Douglas Mental Health University Institute, McGill University, 6875 LaSalle Boulevard, Verdun, Montreal, Québec, H4H 1R3 Canada; Douglas Mental Health University Institute, Montreal, Canada; École de Réadaptation, Université de Montréal, Montréal, Canada

**Keywords:** Training, Eating disorders, Anorexia nervosa, Bulimia nervosa, Scoping review, Healthcare professionals, Educators, Families

## Abstract

This review identified and synthesized published training programs on eating disorders (ED) (anorexia nervosa or bulimia nervosa) for professionals, natural supporters of people with ED, or the public. A scoping review using the Arksey and O’Malley (2005) framework was conducted. Four data bases were searched, for all years, and manual searches from three additional sources were also conducted. Experts on ED were consulted for validation of the identified studies. A narrative synthesis was performed. A total of 20 evaluation studies from five countries were identified, and reviewed in relation to 14 ED training programs. Characteristics of the training programs, and study characteristics, were highly diverse, as shown on Table 1 which compiles results from the charted data. Evaluations were equally divided between training for healthcare and education professionals (9), and training for families or other carers of people with ED (10). One study evaluated ED training for the general public. We found that training orientation varies with the interests and needs of different trainee groups. While most studies assessed trainee outcomes, future research needs to give greater consideration to patient perspectives, and to the relationship between training and evaluation approaches, improved knowledge, and better care.

## Background

This scoping review was conducted to support the development of an evidence-informed training program for primary healthcare providers through the Douglas Eating Disorders Program in Montreal, Canada. The research question was broad: What training programs on eating disorders (ED) are available for professionals, or natural supporters of people with ED? The review aimed to identify, and describe, published training programs that have been both implemented and evaluated. We were interested in identifying training focused on assessment, treatment and support for people with ED, as well as prevention-focused training.

## Method

The scoping review methodology is ideal for rapidly mapping a field of research with a view toward identifying gaps in research or practice. Scoping is usually exploratory, according to Davis et al. [1] The present review used the 6-staged framework for scoping reviews developed by Arksey and O’Malley [2], which is structured in line with a systematic review: development of the research question, study selection, charting, summarizing and reporting results. We also included a consultation stage with experts in the ED field.

### Search procedure

A systematic literature search was conducted using electronic databases, and manual search techniques. Four databases were included: Ovid Medline, Pubmed, Embase, and a keyword search of the Scopus database, using the terms “eating disorders”, “training”, and “primary healthcare”. Date restrictions were not applied, as we were interested in identifying all published training programs on ED. Manual searches for additional studies included: 1) the reference lists of all selected articles; 2) tables of contents for 2009–2014 in the following journals: The International Journal of Eating Disorders; Eating Disorders: The Journal of Prevention and Treatment; European Eating Disorders Review; Eating Disorders; and the Journal of Eating Disorders; and 3) websites for the Academy for Eating Disorders; National Eating Disorder Association (US), and National Eating Disorder Information Canada. Experts on ED were consulted (HS, MI) in order to validate the study selection, and suggest names of other key authors.

### Selection criteria

Inclusion criteria for the study were: 1) published articles in English or French; 2) all study designs; 3) a trainee group: professionals from any discipline; family members or other caregivers of people with ED; the general public; and 4) a target group: people of any age diagnosed, or at risk for, anorexia nervosa or bulimia nervosa. The exclusion criteria were: 1) non-research studies and books, except for descriptions of ED training programs where an evaluation was published separately; and 2) ED intervention studies.

**Fig. 1:**
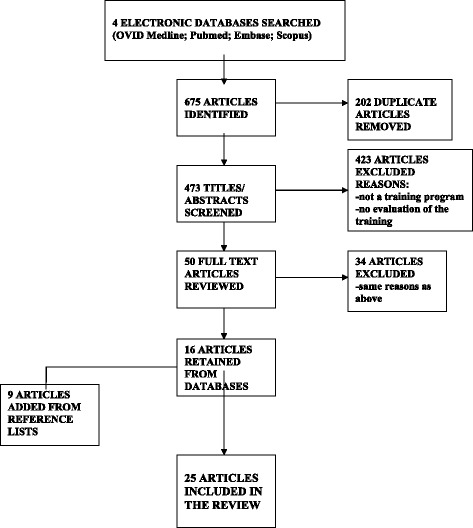
Flow diagram of study selection process

The initial electronic search identified 675 articles, with 473 remaining after 202 duplicates were removed. Titles and abstracts of the 473 articles were independently screened for relevance by two researchers (MP and AP) based on the inclusion and exclusion criteria, and 50 articles selected. The same team members then read the selected articles in their entirety. Thirty-four articles (34/50) were excluded for two reasons: 1) the program described was not a training program; or 2) no evaluation of the training was published. This resulted in a total of 14 articles for the review. Disagreements related to the inclusion of papers were discussed and resolved by involving a third team member (JS). The manual search of reference lists for the 14 selected articles resulted in an additional 6 articles, for a total of 20. No further articles emerged from the secondary searches of ED journals, or the organization websites. It should be noted that additional publications exist describing programs for 5 of the 20 selected articles. While not part of the review, these articles may be consulted for supplemental information [3–7]. Fig. 1 presents a flow diagram for the study selection process.

### Data extraction and synthesis

The research team developed a data charting form consisting of three overall categories: reference information on the studies (title, authors, journal, publication year, author disciplines, country); training program details (program name, objective, approach, setting, training description, trainee and target populations); and details of the evaluations: (purpose, methodology, participants, data collection, study results/outcomes, study limitations/contributions, recommendations). The data were charted by two researchers (AP, JS), in conjunction with the project lead (MP). A narrative synthesis of the data was performed. The narrative synthesis is a conceptual and interpretive approach focused on the relevance and contribution of evidence rather than rigidly determined methodological criteria; it is especially appropriate for synthesizing methodologically dissimilar studies [1, 8].

## Results

### Study characteristics

The 20 evaluation studies reviewed concern a total of 14 ED training programs. The studies represent five countries: Canada (3), UK (5), USA (7), Australia (3), and Norway (2). Table 1 presents the 20 studies in terms of training program identification; training objective/approach; identification of trainee and target populations; evaluation method and findings. The studies included four trainee groups: healthcare professionals (clinicians, students, administrators, and multidisciplinary teams, *n* = 8); educators (teachers, administrators, school staff, *n* = 3); natural supporters (parents or other family members, carers, friends, *n* = 10); and the general public (*n* = 1). Target populations were children and/or adolescents (*n* = 8) or people of all ages (*n* = 14)^1^. For 17 studies the target population had a diagnosed or suspected ED, while the remaining three concerned populations at-risk.

**Table 1:** Characteristics of studies in the review

Training program title	Article ID: Author, year, country	Training objective/approach	Trainees	Target population	Evaluation method	Evaluation findings
Ontario community outreach program for eating disorders	McVey, 2005 [18] (Canada)	Increase community-based practitioners’ knowledge, involvement and level of comfort to treat clients with EDs; to foster linkages among practitioners in and across regions of the province. Based on an evidence-based model of care	Healthcare practitioners; school boards & public health departments	Adults, adolescents, children	Quantitative; pre-post intervention survey	↑ knowledge re ED, body issues; ↑ confidence to treat or teach on ED; better practitioner links
The student body: promoting health at any size	McVey, 2007 [17] (Canada)	A prevention program for elementary school teachers and public health practitioners. The web-based approach made the program accessible both inside and outside school hours	Elementary teachers; public health professionals	Elementary school children	RCT	↑ teacher knowledge re dieting & peer influence; high satisfaction w/ online tools & self as role model
The Meal Support Training (MST)	Cairns, 2007 [28] (Canada)	Introduces concept of meal support; helps others understand feelings of youth with disordered eating around meals; provides approaches/strategies for meal support	Parents, caregivers, friends of eating-disordered youth	Children with ED	Mixed methodology	+ parent ratings on manual & video, especially re patient input. Tools support parental instincts
Maudsley eating disorder collaborative care workshops	Sepulveda, 2008 [26] (UK)	Aims to strengthen knowledge and skills of carers, while reducing the burden of caring for their children with ED. Elements of approach: Skill-based instruction; group format; observation of others’ skills; weekly goals	Family members of people with all forms of ED	Children treated for ED at South London & Maudsley Hospital	Quantitative pre-post design + 3 month follow-up	↓ carer distress and care burden over time; benefits = new skills, exchanging with others
	Sepulveda, 2008a [23] (UK)	Aims to strengthen knowledge and skills of carers, and reduce the emotional burden of caring for their children with ED. Approach includes theory and instruction; demonstration and practice; telephone-administered skills coaching based on behavior therapy	Family members of ED patients	People with ED	Quantitative and qualitative	Quant results not sig. Qualitative: ↑ understanding of how reactions & interactions w/ patients impact outcomes.
Maudsley eating disorder collaborative care workshops (continued)	Macdonald, 2011 (UK)	Aimed at improving communication and reducing social impact of ED for families by addressing negative QOL, burden of illness, distress and expressed emotion. Evidence-based approach, psycho-education principles and motivational interviewing	Family and carers of people with ED	People with anorexia	Qualitative	Skills transfer &supplementary coaching were highly valued; positive change for coaching group & acceptability of intervention
Overcoming Anorexia Online (OAO)	Grover, 2011 [20] (UK)	Aims to provide information, promote self-monitoring and teach skills to identify, understand, and manage Anorexia. Interactive, web-based approach; uses CBT (Williams, 2002, 2009) and systemic framework (Dummett, 2006)	Carers (relatives, partners, friends) of someone with broadly defined anorexia	People with AN, all ages and stages of illness	RCT	Main H: ↓ carer distress after OAO supported (vs. controls). Module on communication was most useful.
The care and understanding of people with eating disorders (ENB N46)	Abuel-Ealeh, 2001 [12] (UK)	Aim of program to raise professionals’ knowledge and awareness of EDs; increase confidence and skills for working with ED clients. A university-level course	Mainly nursing students (1 OT; service users)	People with ED (future clients of trainees)	Quantitative descriptive (some open questions)	81.5 % program completers later worked in ED fields; 77.7 % interested in further training
Collaborative care skills training workshops	Pepin & King, 2013 [22] (Australia)	Replication of the Maudsley eating disorder collaborative care workshops in Australia	Family members	People with ED living with family members	Quantitative pre-post design + follow-up	↑ adaptive coping strategies over time; ↓ over- Involvement (not EE).
	Goodier, 2014 [19] (Australia)	Adaptation of the new Maudsley method for parent skills training with children and adolescents	Parents of children or adolescents in treatment for ED	Children or adolescents with ED	qualitative	Training helped re: managing illness & family dynamics; broke isolation; peer support
Mental health first aid training course for eating disorders	Hart, 2012 [27] (Australia)	Aims to improve mental health literacy in the social networks of individuals with ED; translates the MHFA protocol, which is an action plan that provides information on various mental illnesses to the public, into a program specifically for EDs	General public	Personal contacts (family, friends, classmates etc.) who may need help for ED	Quantitative; pre-post repeated measures design	↑ ED knowledge & first aid strate-gies; ↓ stigma (social distance); ↑ confidence to identify & help someone with ED.
(No title)	Chally, 1998 [16] (USA)	A prevention program for school personnel aimed at providing training to recognize students at risk for ED, or to identify signs and symptoms in students with whom they interact daily	High school educators and staff	High school students potentially at risk for ED	Quantitative, pre-post test, control group design	↑ knowledge & ability to identify students at risk; ↑ belief in getting help; ↓ belief that thin = success.
The eating disorder curriculum for primary care providers	Gurni & Halmi, 2001 [13] (USA)	Aims at providing a first step in training social workers to serve as eating disorder therapists in primary care clinics	9 female social workers	minority group members, low-income, at risk for ED	Quantitative (pilot study)	↑ ED knowledge re assessment & treatment; better diagnostic skills post training.
Group Parent Training program (GPT)	Zucker, 2005 [26] (USA)	Assists caregivers in managing the child’s ED, and facilitates a healthy home environment for sustained change. Draws on narrative family therapy and psycho-educational approaches, emotion-focused therapy, mindfulness strategies, dialectical behavior therapy	Parents/carers of patients in the Duke ED Program	Patients in the Duke ED Program	Qualitative (focus groups)	Parent desire for psycho-education materials w/ skills-based approach; ideas re ↑ peer support.
	Zucker, 2006 [24] (USA)	Overall aim to maximize the effectiveness of parent involvement while minimizing burden in managing EDs; the main approach used dialectical behavior therapy (DBT) adapted to a group parent format. Course content also based on social cognitive, and learning, theories	(No answer)	Adolescent outpatients from the university affiliated medical center	Quantitative descriptive	↑ management of ED, but also better parents; ED skills transfer to other areas; ↑ stress management
Eating disorders and mental health—the EAT framework	DeBate, 2009 [11] (USA)	Aims to increase the capacity of oral health professionals to deliver ED-specific secondary prevention to patients suspected of disordered eating; uses a framework based on transtheoretical model and brief motivational interviewing	Oral health providers	Dental patients suspected of having an ED	Quantitative pre-post design	↑ self-efficacy; ↑ knowledge re oral manifestations of ED, treatments, attitudes re: 2nd-ary prevention
	DeBate, 2012 [10] (USA)	To increase knowledge, skill & self-efficacy among dental and dental hygiene students for dealing with oral manifestations of disordered eating; approach is a theory-based framework based on brief motivational interviewing (B-MI)	Dental and dental hygiene students	Dental patients with signs of disordered eating	Quantitative, group randomized control design	↑ improvement vs controls re ED knowledge, oral findings, skills-based knowledge, self-efficacy
The parent partner program™	Haltom, 2012 [21] (USA)	To provide carers with knowledge and skills to support people with ED, but also bring together a community of professionals, carers and advocates around integrated treatment; uses philosophy of mutual support and learning based on research by Bronfenbrenner (Cochran & Henderson, 1986)	Family, friends caring for ED patients	Anyone with ED	Quantitative pre-post test design	↑ knowledge re ED, treatment; how to provide support, ↑ support re carers & empathy re people w/ ED.
Body and self esteem	Rosenvinge, 2003 [15] (Norway)	Increase clinical competence of health providers in ED; encourage interdisciplinary work at local level, and therapists to as ED resources in health care services; approaches: family therapy; CBT); psychodynamic therapy	Local multi-disciplinary health care professionals	Prospective clients of trainees	Quantitative pre-post design + 1 year follow-up	Needed more time to learn clinical skills, management, therapy; ↑ confidence to treat @ follow-up.
	Pettersen, 2012 [14] (Norway)	Addresses professionals’ needs for clinical competence and better understanding of the benefits of inter-professional collaboration in treating ED; approach is “exchange based”	Doctors, nurses, psychologists & other health care workers	(No answer)	Qualitative	Desire for ↑ ED services & training after program & to work inter-professionally

The evaluation studies employed a heterogeneous mix of study designs: there were 14 quantitative studies (8 pre-post interventions; 3 RCTs; 3 quantitative descriptive studies); another four were qualitative evaluations, and two used mixed methods. The lack of correspondence between study designs, and characteristics of the training programs (e.g. aims, approaches, populations of interest, outcomes) does not allow for numerical pooling of the outcome data [9]. Thus, a narrative synthesis was conducted.

### Training for healthcare and education professionals

Overall, the nine evaluations of ED training for healthcare and education professionals focused on knowledge translation and skill building, prevention, and professional development. Training programs were geared toward specific groups: health care professionals including dentists [10, 11], nurses [12], social workers [13], multidisciplinary health professionals [14, 15], and educators [16–18] . Five evaluations found that training significantly improved trainees’ knowledge, skills, and confidence to assess, treat, or teach on eating disorders [10, 11, 15, 17, 18,]. McVey et al. [18] reported better linkages among ED practitioners, while Rosenvinge et al. [15] documented strong interest in working inter-professionally, or in starting new services, as a result of training.

### Training for families and significant others

Findings from the ten evaluations of ED training for families and other carers also revealed that training improves knowledge and related skills. Yet, most important or this group are findings related to reduced distress and burden, better coping and communication [19–22] and improved family functioning [19, 23]. Trainees needed to be affirmed as good parents, and found that the skills acquired in ED training were transferrable to other areas of parenting [5, 24]. The need for connectedness and support, particularly among parent trainees, emerges as a key theme: being able to break isolation and “externalize the illness” [19]; the need for ongoing exchange with others [19, 25], and extended support through “alumni groups” or “buddy” systems [26].

### Training for the public

Hart et al. [27] evaluated a training program that adapted the Mental Health First Aid (MHFA) protocol for serious mental illnesses to ED. They demonstrated that ED knowledge and helping strategies may be effectively disseminated to the general public. The MHFA training was associated with more accurate recognition of eating disorders, greater knowledge of effective treatments and helping strategies, and confidence in providing help.

## Discussion and conclusions

This review underlines the international scope of interest in ED training, and a more frequent focus on training for people with an ED diagnosis than on prevention. While all the evaluations assessed outcomes for trainees, very few included questions on training effectiveness from the patient perspective; and none controlled for possible confounding influences on training outcomes. Future research is needed to determine the intensity of training required to sustain improvements in ED knowledge and skills. As well, follow-up studies should establish a stronger link between improved knowledge and better care for sufferers. It would also be important to develop training fidelity measures.

The results suggest that the orientation of ED training varies with the interests and needs of different trainee groups. Whereas healthcare professionals and educators are concerned with the overall development of the ED field, and dissemination of best practices, training for the public at-large promotes familiarity with ED and actual contact between ordinary citizens and ED patients, addressing the critical issues of social distance and stigma in mental health populations [28, 29]. Moreover, family involvement with ED is particularly intense and personal, identifying them as not only trainees, but a potentially vulnerable target group. Results suggest that the supportive, face-to-face element of training for families and natural supporters, both between trainers and trainees and among trainees themselves, was highly beneficial. This implies that ED training using passive learning approaches may be less effective for families, for whom the lived experience of training was an added value.

The heterogeneity of these studies, divergent objectives of the training programs, and the wide array of methodologies employed precluded a more in-depth comparison of individual studies, or subgroups. Nonetheless, the review does provide a comprehensive overview of research on ED training initiatives that should be of interest to healthcare practitioners, educators, and families involved with the management or prevention of ED, as well as the interested public.

## Endnote

^1^The number of studies reported here totals 22, instead of 20. This occurred because two studies, McVey, 2005, and 2007 cut across two professional groups (healthcare professions and educators), and both age groups (children, and all ages), so are counted twice.
